# Medical Emergency Equipment Medication (MEEM) Training: A Quality Improvement Project Focusing on Transforming the Emergency Response to Inpatient Psychiatric Medical Emergencies

**DOI:** 10.1192/bjo.2024.438

**Published:** 2024-08-01

**Authors:** David Prossor, Simona Constantinescu, Cem Stubbs, Neil Stewart, Fredrik Johansson

**Affiliations:** 1 East London NHS Foundation Trust, London, United Kingdom; 2 Barnet, Enfield and Haringey NHS Trust, London, United Kingdom; 3 Camden and Islington NHS Trust, London, United Kingdom

## Abstract

**Aims:**

All medical staff working within NHS psychiatric hospitals in the UK are required to complete mandatory life support training. However, there is no such mandatory requirement for associated training around the effective use of the emergency medical equipment used during medical emergencies on inpatient psychiatric wards. This quality improvement project focused on developing a sustainable educational intervention aimed at all staff types within one London inpatient psychiatric hospital. Staff of all grades and roles encountered frequent difficulties and delays in relation to the emergency medical bags and equipment, including issues around skill and confidence.

**Methods:**

A survey was initially sent to medical and nursing staff working on an inpatient psychiatric unit, which highlighted participants’ lack of confidence in using the equipment. It emerged that staff exclusively handled the emergency medical equipment during relatively rare emergencies. This resulted in unfamiliarity with the equipment and consequent difficulties in using it competently. A novel educational intervention dedicated to upskilling staff with emergency medical equipment was created, focusing on contents and use of individual equipment within the medical emergency bag. Pre- and post-intervention quantitative feedback regarding confidence and familiarity was obtained using feedback forms containing Likert scales. Qualitative feedback was also obtained.

**Results:**

More than six training cycles, each consisting of at least five training sessions, have now been completed with both qualitative and quantitative measures of improvement captured. Individuals noted on average a 31.62% (±3.605%) improvement in self-reported confidence and familiarity with equipment. The most frequently identified positive themes were that the intervention familiarised staff with equipment and was educational, whilst the most frequent suggestion for improvement were requests for additional sessions. From single idea to sustainable quality improvement, the team broadened and gained stakeholder support including clinical and nursing directors, pharmacy, junior doctors, nurses, and matrons.

**Conclusion:**

The intervention has achieved sustainability and is being explored in other partnership psychiatric hospitals. Despite reported increased confidence in handling the emergency equipment, there is ongoing need to develop, maintain and practice these skills, across both the nursing and medical staff, to achieve better outcomes for psychiatric inpatients. Trainee psychiatrists intend to develop the project further, and the training will be incorporated as a mandatory requirement. The project links to the quality standards for mental health point 12 of the Resuscitation council UK. Next stage developments of the project include linking to feedback from emergencies as well as incorporating into existing simulation training.

